# Robust time delay estimation for measurement of ictal EEG traveling waves

**DOI:** 10.1088/1741-2552/ae9bed

**Published:** 2026-09-04

**Authors:** Trisha Mendoza, Marco Antonio Pinto-Orellana, Joffre E Olaya, Hernando Ombao, Daniel W Shrey, Beth A Lopour

**Affiliations:** 1 Department of Biomedical Engineering, University of California, Irvine, CA, United States of America; 2 Division of Neurosurgery, Children’s Hospital of Orange County, Orange, CA, United States of America; 3 Department of Neurosurgery, University of California Irvine, Irvine, CA, United States of America; 4 Statistics Program, King Abdullah University of Science and Technology, Thuwal, Saudi Arabia; 5 Division of Neurology, Children’s Hospital Orange County, Orange, CA, United States of America; 6 Department of Pediatrics, University of California, Irvine, CA, United States of America

**Keywords:** epilepsy, seizure, ictal wavefront, source localization, intracranial EEG, humans

## Abstract

*Objective.* Seizures are often characterized by epileptiform discharges that appear to be highly synchronous across electroencephalogram (EEG) channels, yet closer analysis reveals small time delays indicative of fast traveling waves. Microelectrode studies suggest that a possible source of these waves is the ictal wavefront, a slowly advancing boundary emitting opposing waves; attempts to extend this analysis to macroelectrodes produced conflicting theories, suggesting both static and moving radial sources. Because variability in EEG measurement and analysis may contribute to conflicting results, we systematically evaluated how signal characteristics and methodological choices affect wave direction estimates. We applied the resulting framework to human intracranial EEG (iEEG) to validate the results. *Approach.* We simulated iEEG data using a second-order autoregressive model, modeling coherence as a distance-weighted sum of signals. We then applied one of eight different time-delay propagation patterns at a range of wave speeds, and accuracy was assessed for multiple referencing schemes, sampling rates, and spatial resolutions. Human iEEG recordings were then analyzed to validate methodological recommendations and characterize ictal waves. *Main Results.* Simulations revealed that wave patterns could be measured most accurately for wave speeds <1000 mm s^−1^ and average iEEG coherence levels >0.4. Under these conditions, a corner electrode reference outperformed other referencing schemes. Wave estimation accuracy was highest for high sampling rates and low electrode spacing. Accurate results were obtained at 3 mm electrode spacing, but not at 9 mm spacing, which approximates standard clinical subdural grids (*p* < 0.01). Human iEEG exhibited a wide range of propagation patterns, including spirals, sources, and sinks which have not been previously reported. *Significance.* Detailed analyses of simulated and human iEEG data highlight critical methodological choices that must be considered when characterizing complex seizure wave patterns. Our findings provide a validated framework to increase the rigor of future studies.

## 1. Introduction

During a seizure, the intracranial electroencephalogram (iEEG) recording is often characterized by high-amplitude spike-wave discharges, which may appear hypersynchronous. However, detailed spatiotemporal analyses reveal that these discharges are rapidly traveling waves propagating across the cortex [1–3]. The origin of these waves is thought to be linked to seizure-generating regions in patients with refractory epilepsy, motivating significant efforts to quantify their dynamics and localize potential sources. Studies using standard iEEG electrodes, which we will refer to as ‘macro’ scale recordings, have shown that these fast-traveling waves can be emitted by a static or slowly moving source that radiates activity outward at speeds ranging from 100 to 1000 mm s^−1^ [1, 2]. In contrast, microelectrode recordings suggest that seizures propagate via a slowly advancing boundary known as the ictal wavefront [4–7]. This wavefront, not observed at the macro scale, is characterized by sustained bursts of high gamma activity in the range of 80–150 Hz and propagates at approximately 1 mm s^−1^ [4, 8]. It has been theorized that this slowly advancing wavefront emits fast-traveling waves in opposing directions, further depolarizing the surrounding tissue and recruiting it into the seizure [4, 8]. More recently, it has been suggested that complex patterns of ictal traveling waves could be generated by a combination of both static and moving radial source(s) and the ictal wavefront [3]. Note that these wave-like phenomena are distinct from fast oscillations in iEEG (high frequency oscillations, ripples, or fast ripples), which can co-occur with epileptiform discharges, have a strong association with the seizure onset zone (SOZ) [9–11] and can also exhibit propagation patterns [12, 13].

Our inability to reach a unified theory regarding the source of these traveling waves could be due to three categories of factors. First, the EEG electrode configuration defines the spatial scale for quantifying these fast-traveling waves [1–3]. Microelectrodes (80 *µ*m diameter, 400 *µ*m spacing) provide exceptional resolution of sub-centimeter phenomena, such as the ictal wavefront. However, they often do not capture seizure onset and cannot track wave propagation across cortical regions [4, 7]. Therefore, ictal propagating waves have been studied with macroelectrode recordings (2.4 mm diameter, 10 mm spacing) to analyze broader cortical regions [1, 2]; however, macroelectrodes produced noisier estimates and could not resolve smaller phenomena such as the ictal wavefront [2]. The use of these disparate spatial scales has hindered efforts to harmonize micro- and macro-scale phenomena.

Second, iEEG analysis methods, such as the referencing scheme, sampling rate, and algorithm for delay estimation, can significantly impact the characterization of these waves. Calculating wave speed and direction relies on an estimation of time delays between the EEG at adjacent electrodes, and the various algorithms exhibit distinct trade-offs. The max descent algorithm provides high temporal resolution for wave direction estimation but tends to produce noisier results [3, 4]. In contrast, the group delay algorithm uses a coupling measure to produce robust estimates even when the signal-to-noise ratio is low, but with lower temporal resolution [1, 3, 14]. The choice of EEG referencing scheme can also directly affect these measures [15–18]. Lastly, selecting a proper sampling rate is critical, as higher rates may increase accuracy of time delay estimates and avoid aliasing.

Third, our ability to accurately quantify traveling waves is impacted by characteristics inherent to the iEEG data, such as the propagation pattern, wave speed, and coherence. Ictal discharges are associated with various complex propagation patterns, with planar wave activity being the most common [1]. The faster these waves propagate, the more difficult they are to accurately measure. Moreover, coherence between iEEG signal pairs reflects the underlying signal-to-noise ratio, which influences the accuracy of wave propagation estimates; however, iEEG coherence depends on the choice of reference electrode [16, 17, 19]. Emphasis is often placed on selecting a ‘quiet’ reference, independent of the propagating wave, as high amplitudes in a common reference signal can lead to spuriously high coherence estimates and distort the phase of the signal [16, 20]. This phase distortion can compromise time delay measurements and subsequent estimates of wave propagation.

Therefore, here we use simulated iEEG as a controlled environment to test both modifiable factors and those inherent to the data. We varied signal characteristics, such as coherence levels, propagation patterns, and wave speeds, and evaluated their interactions with methodological choices including referencing schemes, sampling rates, and grid sizes. Using this framework, we identified the advantages and disadvantages of various methodologies and determined thresholds that yielded the most robust wave direction estimates. Once these guidelines were established, we applied them to human iEEG data to demonstrate efficacy in a real-world application. Our findings highlight the importance of assessing signal characteristics that are beyond experimental control, such as coherence, as they can inform methodological decisions to maximize measurement accuracy. Moreover, we provide practical guidelines for future analyses of traveling waves by identifying factors that are critical for accurate results.

## 2. Methods

### 2.1. Wave direction estimation and propagation pattern classification

For both our simulated and human iEEG, we estimated wave direction and speed using the group delay method described previously [1, 3, 14]. We chose this method because we wanted to prioritize the accuracy of the estimates, as opposed to the temporal resolution. For an 8 × 8 grid of electrodes, we computed the 1–13 Hz cross-spectrum and magnitude-squared coherence between the electrode of interest and up to two surrounding electrodes on each side. Thus, electrodes in the center of the grid were compared to their surrounding 5 × 5 subgrid, and corner and edge electrodes were compared to the subset of existing electrodes within a surrounding 5 × 5 subgrid (a minimum of eight for corner electrodes). Coherence was used as a means to threshold our cross spectrum values; specifically, we identified frequency intervals of ⩾3 Hz width in which coherence exceeded the 95% theoretical confidence level [1]. If there were multiple intervals of equal width, the interval with the higher average coherence was selected. The cross-spectrum values corresponding to this interval of high coherence were then extracted and a linear regression was applied. The independent variable consisted of the frequency values in the interval and the dependent variable was the corresponding cross spectrum values, which enabled calculation of the time delay (the slope) between neighboring electrodes and the central electrode. If the electrode pairs did not meet the significance requirement for coherence (no intervals ⩾3 Hz width) or the linear regression did not obtain a significant fit (*p* < 0.05), no delay was calculated.

We then arranged the time delays according to their physical (*X, Y*) positions in the electrode grid, and we fit a plane $\Delta \hat t = {b_0} + {b_1}X + {b_2}Y$ to these time delays by estimating parameters ${b_0},{\text{ }}{b_1},$ and ${b_2}.$ The fit was considered significant if: (1) at least 50% of the subgrid electrodes had estimated delays relative to the central electrode, and (2) the fit for this model produced significantly lower mean squared error (MSE) than the null model where ${b_1} = {b_2} = 0$. (*F*-test, *p* < 0.05). If both criteria were satisfied, we estimated wave speed as $1/\sqrt {b_1^2 + b_2^2} $, and wave direction as $ta{n^{ - 1}}( {\frac{{{b_2}}}{{{b_1}}}} )$. If the criteria were not met, wave speed and direction were not estimated for that electrode. This analysis was conducted on 10 s windows of EEG with a 9 s overlap.

**Figure 1.**
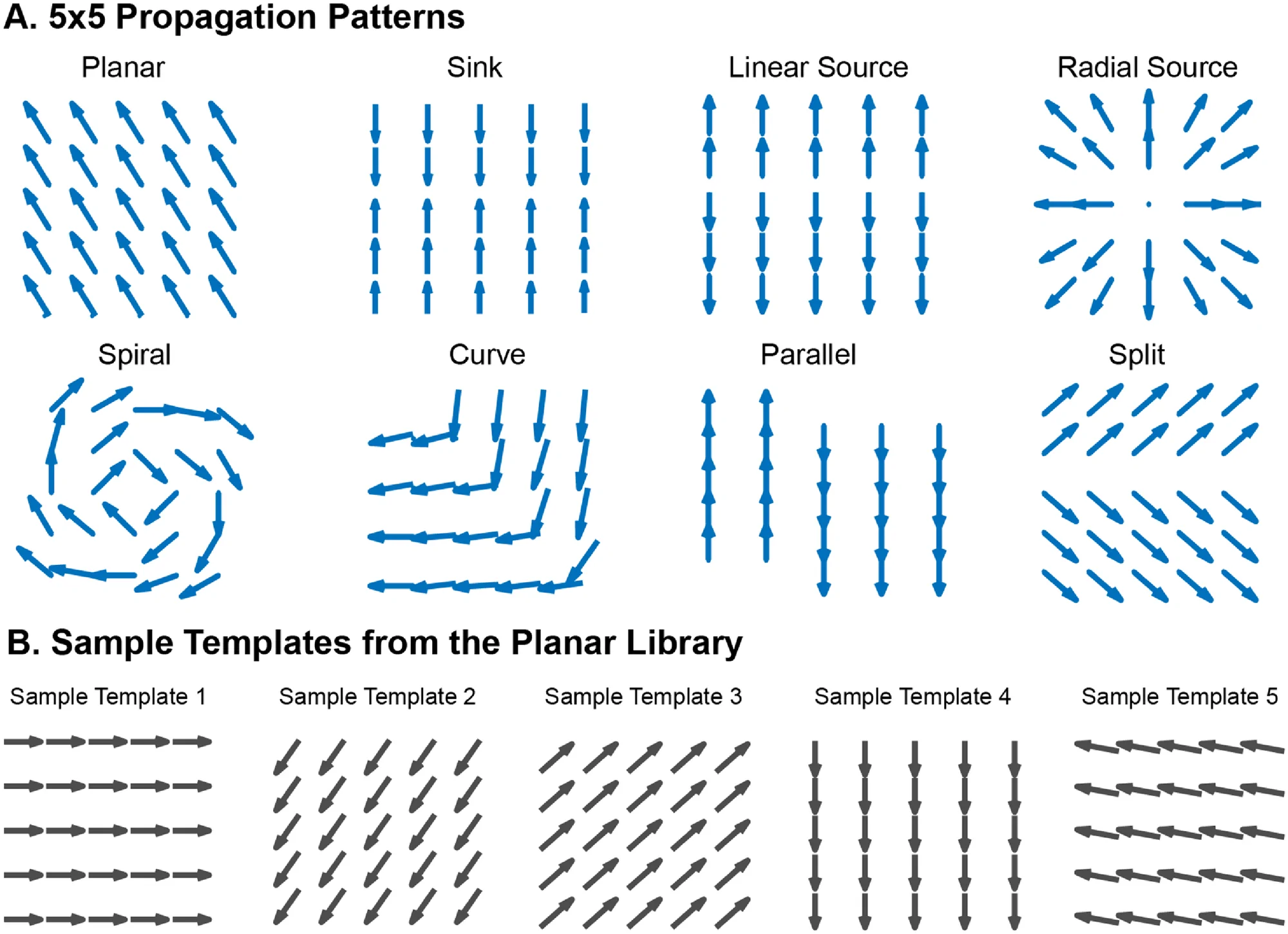
Sample 5 × 5 templates for pattern clustering. (A) We used eight patterns to classify local wave directions in each 5 × 5 subgrid contained within the larger 8 × 8 grid. The arrows represent the wave direction at each electrode, computed using the coefficients of the plane fit to the time delay estimates for the surrounding subgrid. One example of each wave propagation pattern is shown. (B) To account for varying directions and locations, a library of templates was developed for each wave propagation pattern, e.g. a set of planar waves, where each wave pointed in a different direction. Here, five sample templates from the planar wave library are shown.

Once wave direction estimates were completed for all electrodes in the 8 × 8 grid, we implemented topological pattern matching to categorize the various propagation patterns seen in the data. To account for situations in which multiple patterns were present or the patterns were not centered on the 8 × 8 grid, we completed this matching process for each 5 × 5 sub-grid contained within the 8 × 8 grid. For each propagation pattern seen in our iEEG data (planar, sink, linear source, radial source, spiral, curve, parallel, and split waves), we developed a library containing templates of each main pattern rotated at various angles (figure 1).

Then the MSE was calculated between all templates and the measured wave directions for one 5 × 5 subgrid. This was repeated for all subgrids that contained at least 12 estimated wave directions. Each subgrid was labeled as one of the eight patterns, based on the template with the lowest MSE, and those with less than 12 wave direction estimates or MSE > 2 were categorized as noise/unknown. Therefore, at each point in time, the 8 × 8 electrode grid was labeled with up to 16 wave patterns, one for each 5 × 5 subgrid.

### 2.2. Accuracy of wave pattern classification in simulated iEEG

#### 2.2.1. Simulating grids of iEEG data with specified coherence levels and time delays

We simulated broadband iEEG data as the sum of five bandpass signals in the delta (0.4–4 Hz), theta (4–7 Hz), alpha (8–12 Hz), beta (12–30 Hz), and gamma (30–50 Hz) frequency bands. To create the narrowband signals ${x_k}\left( t \right)$, we used a second-order autoregressive model, denoted as AR(2) [21, 22]. The AR(2) model generates signals through a stochastic process that combines a linear function of the two preceding points with an added Gaussian white noise term ${\varepsilon _k}\left( t \right)~WN\left( {0,\sigma _e^2} \right)$:

\begin{equation*}{x_k}\left( t \right) = {\phi _{1k}}{x_k}\left( {t - 1} \right) + {\phi _{2k}}{x_k}\left( {t - 2} \right) + {\varepsilon _k}\left( t \right).\end{equation*}

Here the coefficients, ${\phi _{1k}}$ $ = \frac{2}{{{M_k}}}\cos \left( {2\pi \times {\Omega _k}} \right)$ and ${\phi _{2k}} = - \frac{1}{{M_k^2}}$ determine the bandwidth and peak frequency of each narrowband signal [22, 23]. The variable $\Omega _k$ is defined as the central frequency for the band of interest (${{{\bar \Omega }}_k})$ shifted by a small random perturbation ${\delta _1}\sim U\left( { - a,a} \right)$, where $a = 0.3{\text{ Hz}}$, to introduce variability across simulations, and normalized by the sampling rate: ${\Omega _k} = \frac{{\left( {{{\bar \Omega }_k} + {\delta _1}} \right)}}{{fs}}$. To ensure causality, the parameter ${M_k}$ (which is the magnitude of the root of the AR(2) polynomial function) must be greater than 1 and is defined as: ${M_k} = {\text{ }}{e^{{m_k}}}{\text{ }}(m &gt; 0)$. All values of ${\bar \Omega _k}$ and ${m_k}$ which correspond to the traditional delta, theta, alpha, beta, and gamma bands, are listed in supplementary table 1.

**Table 1.** Characteristics of all eight simulated coherence levels. The mean, median, and IQR were derived from coherence values computed between each of the 64 channels and the center-most electrode. For our experiments, coherence levels 3, 6, and 8 represent low, medium, and high coherence, respectively.

Coherence level	1	2	3 (Low)	4	5	6 (Med)	7	8 (High)
Mean	0.23	0.26	0.30	0.35	0.43	0.53	0.65	0.71
Median	0.21	0.24	0.28	0.33	0.40	0.50	0.62	0.68
IQR	0.18–0.26	0.2–0.29	0.23–0.33	0.27–0.38	0.35–0.47	0.44–0.58	0.56–0.71	0.62–0.78

The magnitude of each component ${x_k}\left( t \right)$ was weighted such that power decreased with frequency by 1/*f* to simulate the characteristic power spectrum of iEEG data, and the components were summed to create the simulated broadband signal $z\left( t \right):$

\begin{equation*}z\left( t \right) = \mathop \sum \limits_{k = 1}^5 \frac{1}{{{{{\Omega }}_k}}}{x_k}\left( t \right) + v\left( t \right),\end{equation*}

where $v\left( t \right){\text{ }}$ is a white noise process independent of each of the oscillatory components ${x_k}\left( t \right),{\text{ }}k = 1,2,3,4,5.$

**Figure 2.**
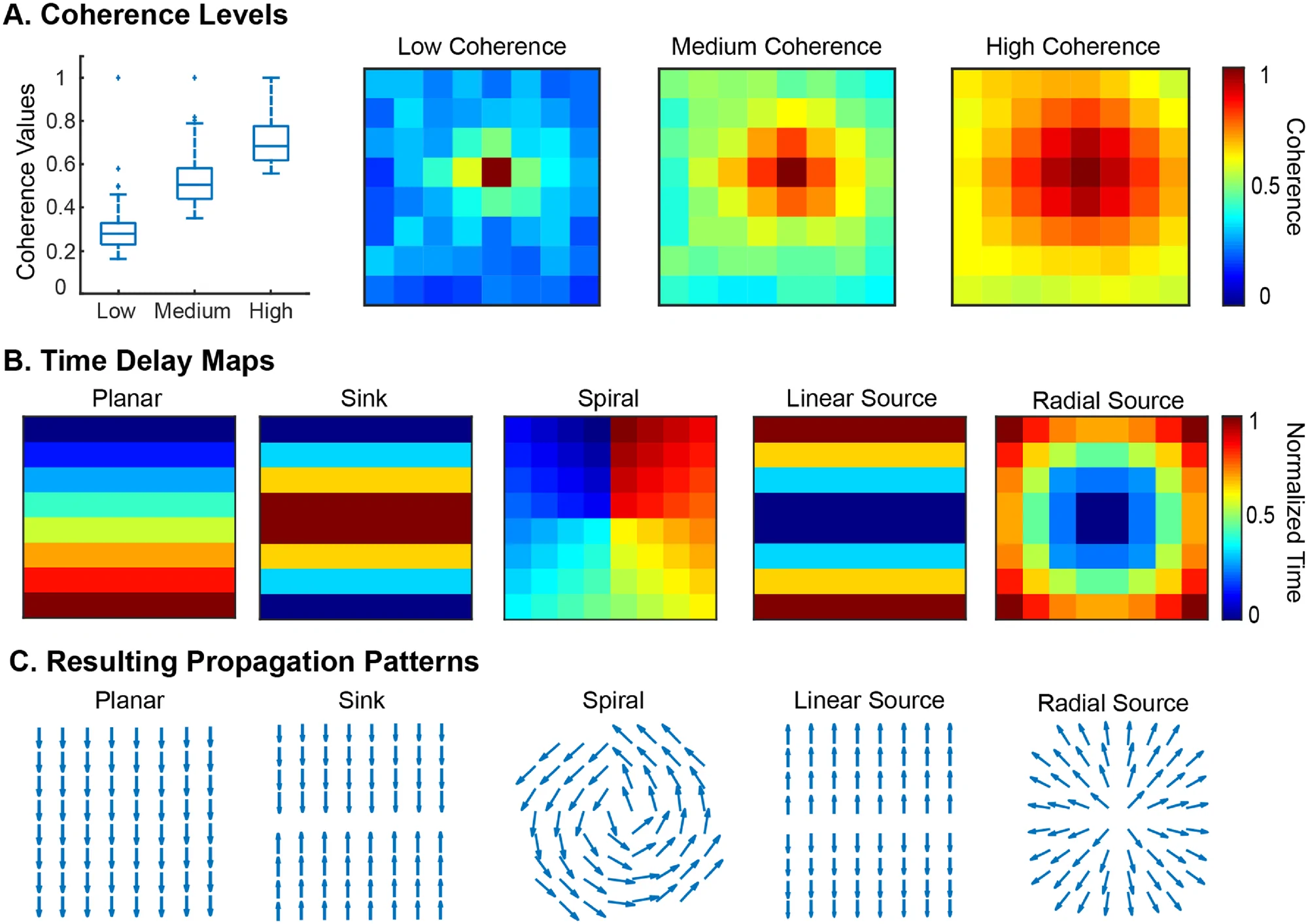
Propagation patterns and coherence levels for wave simulations. (A) Eight coherence levels were used to simulate the iEEG signals in the 8 × 8 grid; the parameters used to generate the coherence levels are shown in table 1. Here we show the levels designated as low, medium, and high coherence, corresponding to levels 3, 6, and 8, respectively. The subfigure on the left shows the distributions of coherence values across the grid for low, medium, and high coherence levels. The three subfigures on the right show example heatmaps of coherence relative to a central electrode. (B) Maps representing the 8 × 8 grid of time delays (τ) added to the signals ${y_i}\left( t \right)$ to obtain the wave propagation patterns. The color represents the time delay, with the largest time delays shown in red. For the purposes of visualization, each delay map was normalized by dividing each delay value by the maximum delay, preserving the relative spatial distribution while scaling all values to the interval [0, 1]. (C) The five wave propagation patterns used to generate simulated EEG signals in our study. Each arrow represents the wave direction calculated at one electrode using the estimated time delays. Abbreviation: iEEG = intracranial EEG.

We generated 64 channels of broadband data ${z_j}\left( t \right)$ to simulate an 8 × 8 grid of EEG electrodes. To prescribe the coherence relationship between electrodes i and j in the grid, ${z_j}\left( t \right)$ was first filtered between 1 and 13 Hz, as this was the frequency band of interest in prior studies of ictal waves [1, 3]. Let $\omega = \left[ {1,13} \right]{\text{ Hz}}$, where ${z^\omega }\left( t \right)$ represents the signal with power isolated in band ω Different coherence levels for each pair of signals were simulated by varying a weight ${A_{ij}}$, determined as the inverse of the Euclidian distance between the electrodes. Specifically, small interelectrode distances simulated high coherence scenarios and larger distances simulated lower coherence levels (figure 2(A)). Horizontal and vertical distances were represented by ${d^x}$ and ${d^y}$, respectively:

\begin{equation*}{A_{ij}} = \frac{1}{{\sqrt {{{\left(d_j^x - d_i^x\right)}^2} + {{\left(d_j^y - d_i^y\right)}^2}} }},{ }i \ne j\end{equation*}

For i=j, we set ${A_{ij}} = 1$. The weighted, filtered signals were then summed:

\begin{equation*}{y_i}\left( t \right) = \mathop \sum \limits_{j = 1}^{64} {A_{ij}}z_j^\omega \left( {t + {\delta _2}} \right).\end{equation*}

Here ${\delta _2}\sim U\left( { - 5,5} \right){\text{ ms}}$ randomly shifts the signal in time to mitigate any artificially high synchrony caused by the summation of signals with a spatial relationship. The resulting signal coherence, developed from varying interelectrode distances, was measured to extract the associated means, medians, and interquartile ranges at each level, which are summarized in table 1.

Finally, various 8 × 8 time delay maps (matching the size of our simulated electrode array) were developed for the following patterns: planar, linear source, radial source, linear sink, and spiral (figure 2(B)). These patterns were created to mimic propagation patterns possible in human ictal iEEG data (figure 2(C)). By increasing or decreasing the delay time between channels, relative to the distance between electrodes, we could simulate varying wave speeds for each pattern. Once a map and corresponding wave speed were selected, time delays were inserted by shifting each signal, ${y_i}\left( t \right)$, by its corresponding channel delay, τ. This 1–13 Hz signal ${y_i}\left( {t + \tau } \right)$, which had prescribed coherence and time delay relationships, then replaced the filtered uncorrelated signal ${z^\omega }\left( t \right)$ in $z\left( t \right)$ to create the broadband, simulated iEEG signal $z^{\prime}\left( t \right)$. Mathematically, $z^{\prime}\left( t \right) = z\left( t \right) - {z^\omega }\left( t \right) + {y_i}\left( {t + \tau } \right)$.

#### 2.2.2. Quantification of wave pattern classification performance

To quantify our ability to accurately classify simulated wave patterns using the estimated time delays, we first categorized the time delays of all 5 × 5 subgrids (*n* = 16) within our 8 × 8 simulated grid using topological pattern matching (section 2.1). During the matching process, we expected the template with the lowest error to be the one matching the propagation pattern originally used to simulate the grid of signals (figure 2). Based on this, we calculated the classification performance as the percentage of subgrids that matched the pattern used to generate the simulated iEEG, minus a penalty term to account for electrodes that did not exhibit a significant wave direction:

\begin{align*}&amp; {\text{classification performance}} = \nonumber\\ &amp;\quad\frac{{\# {\text{true positives}}}}{{{\text{total subgrids}}}} - \alpha \cdot \left( {\# {\text{electrodes with missing estimate}}} \right).\end{align*}

Here *α* = 1/64, so the classification performance equals −1 when no electrodes yield a significant wave direction, and it equals 1 when all electrodes have an estimated wave direction and all subgrids are correctly classified. This classification performance calculation was used on all simulations except grid spacing, due to varying grid sizes.

#### 2.2.3. iEEG signal properties that impact wave estimation: coherence and wave speed

We hypothesized that lower coherence and higher wave speeds would result in noisier estimates of wave directions and lower pattern classification performance. Therefore, we systematically tested every propagation pattern for wave speeds of 100, 300, 500, 700, 900, and 1000 mm s^−1^, chosen to match previously reported values in humans [1]. For all propagation patterns and wave speeds, we also tested eight levels of coherence (table 1).

#### 2.2.4. Methodological choices that impact wave estimation: referencing, sampling rate, and grid spacing

**Referencing Schemes:** To investigate how different EEG referencing schemes affect wave direction estimates, we first generated 8 × 8 grids of iEEG data with low, medium, and high coherence levels (levels 3, 6, and 8 respectively, figure 2(A)). We then simulated planar, source (radial and linear), sink, and spiral wave patterns (figure 2(C)) at three wave speeds (100, 500, and 1000 mm s^−1^). Then we applied three referencing schemes to the 64 iEEG channels: a common average reference, a single electrode reference selected from the corner of the grid, and a single, distant electrode reference. In the simulations, the distant signal was generated using an AR(2) model as an independent broadband iEEG signal, designed to have no 1–13 Hz coherence relationship with any of the 64 iEEG channels, which themselves shared a structured coherence relationship. Wave directions were then computed at each electrode (see section 2.1), and the classification performance was calculated for 30 repetitions of the simulation.

**Sampling Rate:** Higher sampling rates may improve wave direction estimation by capturing smaller time delays, but they are computationally expensive. Therefore, we tested the classification performance as sampling rate and wave speed varied. We first simulated a grid of iEEG with high coherence and a 5000 Hz sampling rate, then progressively downsampled the signals five times to produce sampling rates of 2500 Hz, 1250 Hz, 1000 Hz, 625 Hz, and 500 Hz. We tested all propagation patterns (figure 2(C)) at five wave speeds: 100, 300, 500, 700, and 1000 mm s^−1^. We then computed the classification performance for all parameter combinations, and each combination was simulated 30 times to assess variability.

**Grid Spacing:** For the tests of grid spacing, we began with a dense 20 × 20 electrode array with contacts spaced 3 mm apart. Channels were simulated with level 8 coherence (high) and a wave speed of 500 mm s^−1^ across all propagation patterns. The electrode grid was then progressively subsampled to simulate arrays with 6 mm and 9 mm spacing. Note that the 9 mm configuration reflects spacing similar to standard macroelectrode subdural grids for clinical recordings.

Due to varying grid sizes, accuracy was calculated using the MSE between the true pattern and the estimated pattern for each simulation. To account for regions without estimated wave directions, values corresponding to non-significant electrodes were set to zero. Therefore, as the number of electrodes with significant direction estimates decreased, the MSE approached 1. Wilcoxon signed rank tests were performed to determine significant differences between errors for all simulated grid configurations.

### 2.3. Human iEEG analysis: data collection & wave pattern classification

Collection of human data for this prospective study was conducted in accordance with the principles embodied in the Declaration of Helsinki and approved by the Institutional Review Board of the Children’s Hospital of Orange County. Informed consent for participation in the research study and publication of the results was obtained prior to patient involvement. During phase 2 presurgical invasive monitoring, three patients diagnosed with drug-resistant focal or lesional epilepsy were implanted with a high-density (HD) 8 × 8 subdural grid of intracerebral EEG electrodes (Ad-Tech FG54C-MP03X-000). The HD grids were implanted in patients that required monitoring with subdural grids for their surgical evaluation, as determined clinically by the epilepsy team. To be included in this study, patients were required to have one of the following: (1) The presence of a lesion on MRI that was believed to be involved in seizure generation, as determined by the patient’s phase 1 workup; or (2) Prior stereoelectroencephalography (SEEG) monitoring that localized the seizure onset to a sub-lobar region. In all cases, both standard subdural grids and the HD grid were used to further localize the SOZ and/or map cortical function to aid in surgical planning. The details of each patient’s ictal onset, epilepsy type, pathology, and resection can be found in supplementary table 2.

Each electrode in the HD grid had a surface area of 1.08 ${\mathrm{m}}{{\mathrm{m}}^2}$ with 3 mm center-to-center spacing, and the grid was placed over the suspected SOZ, based on available clinical data. Additional grids, strips, and depth electrodes were implanted as determined necessary by the clinical team; however, here we only analyze data from the HD grid. iEEG data sampled at 5000 Hz were recorded from each patient across several days, with the goal of capturing multiple seizures. For our analysis, one representative seizure was selected for each patient (three seizures total). Seizure start times were visually determined by a board-certified pediatric epileptologist, and termination times were determined as the latest time the HD subdural grid displayed large amplitude ictal fluctuations [1]. Data were clipped to include 10 s prior to seizure initiation and 10 s after termination.

We performed time delay estimation and wave pattern classification on these clips of human iEEG to confirm that they were consistent with our simulation results. For each seizure, we calculated the wave directions with the group delay method (section 2.1; 10 s windows with 9 s of overlap) using three distinct referencing schemes: (1) We chose an electrode that was not recruited during the seizure or not exhibiting typical ictal low frequency large amplitude fluctuations, to serve as a distant, single electrode reference. (2) We used a corner electrode from the HD subdural grid. (3) We applied a common average reference using the 64 channels of the HD subdural grid. The resulting pattern classifications were plotted for the entire duration of the seizure for direct comparison between the three referencing methods.

To assess the impact of sampling rate on wave direction estimation in human iEEG, we first calculated the wave directions using the original sampling rate of 5000 Hz and classified the resulting wave patterns across all seizures. We then progressively downsampled the original signal to 500 Hz. At each downsampling step, we estimated the wave directions and classified the resulting wave patterns across the entire seizure to determine how the sampling rate influenced the results.

## 3. Results

### 3.1. Wave direction estimates are most accurate for high coherence levels and low wave speeds

**Figure 3.**
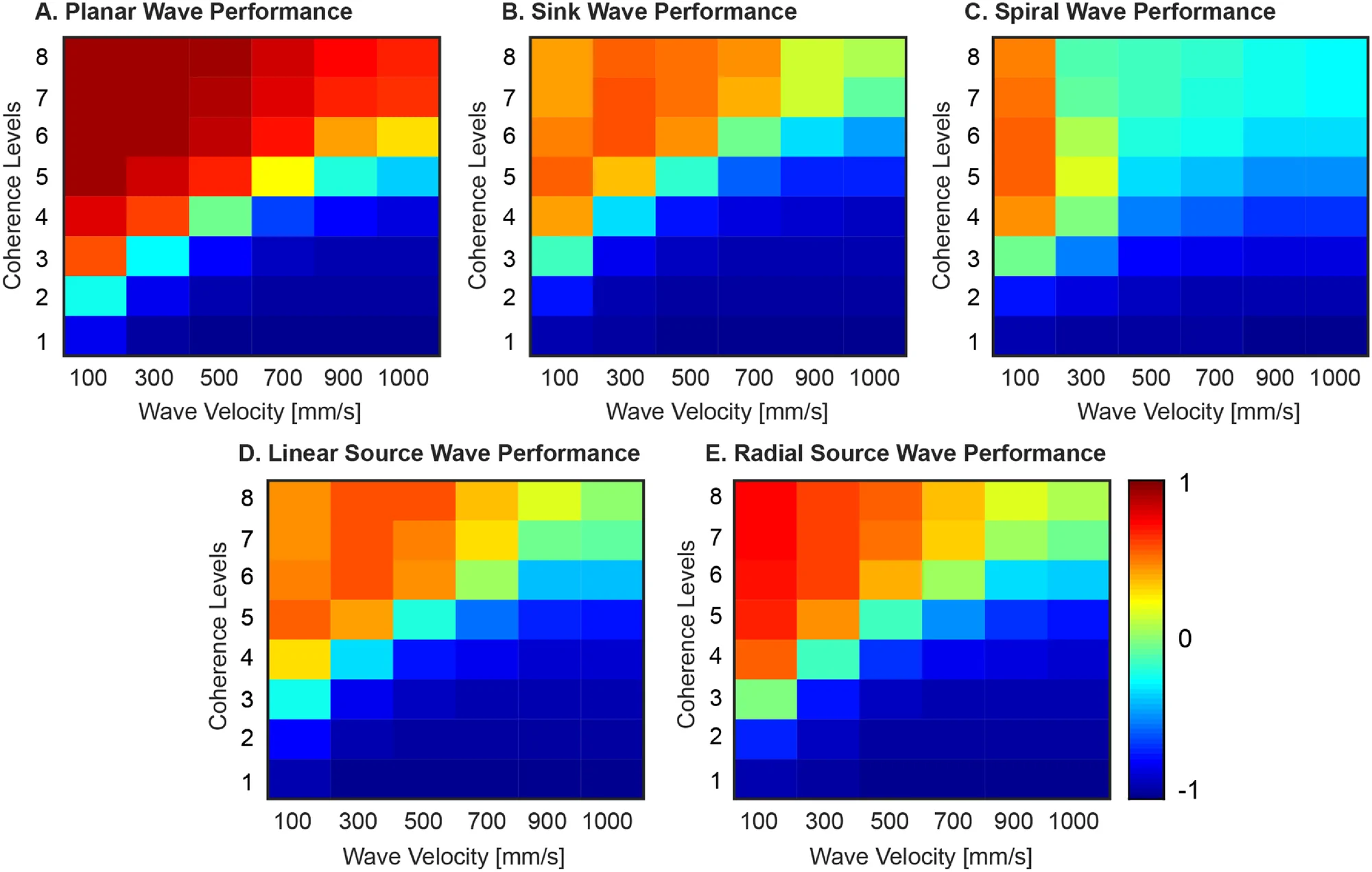
Wave pattern classification performance is highest for high coherence and low wave speeds. The average classification performance values for (A) planar, (B) sink, (C) spiral, (D) linear source, and (E) radial source waves are shown as a function of coherence levels (table 1) and wave velocities for all tested patterns. Averages were calculated across 30 iterations of each simulation, and heatmaps of the IQR values for each wave pattern are shown in supplementary figure 1. Low wave speeds and high coherence allow for the most accurate classification across most of the propagation patterns, while spiral waves are captured accurately under wave speeds around 100 mm s^−1^ and higher coherence levels. Abbreviation: IQR = interquartile range.

Our results show that both wave speed and coherence influence the performance of wave pattern classification (figure 3, supplementary figure 1). For all pattern types, the classification performance decreases as wave speed increases, requiring higher levels of coherence in order to correctly classify the wave pattern.

However, the overall level of performance depends on the pattern type. While most of the patterns show a range of coherence and wave speed values that allow for accurate classification, spiral waves are only accurately detected at low wave speeds (100 mm s^−1^; figure 3(C)). Recall that a classification performance close to 0 (green) indicates potential noise or misclassifications in the wave direction estimates, while values of −1 (dark blue) indicate an inability to detect wave directions within the subgrids. In contrast to spiral waves, planar waves were less susceptible to the effects of high wave speeds, as they could be accurately detected at any speed if the signals were sufficiently coherent (figure 3(A)). Linear source, radial source, and sink wave patterns have similar parameter ranges in which classification is accurate, but overall, they are slightly more difficult to measure than planar waves (figures 3(B), (D) and (E)). The choice of referencing scheme did not significantly impact these results (supplementary figure 2).

### 3.2. Referencing schemes disrupt propagation patterns, especially for low coherence and high wave speeds

To evaluate how different referencing schemes affect wave direction estimates, we applied distant electrode, corner electrode, and common average references to the simulated iEEG and assessed the classification accuracy for each one.

**Figure 4.**
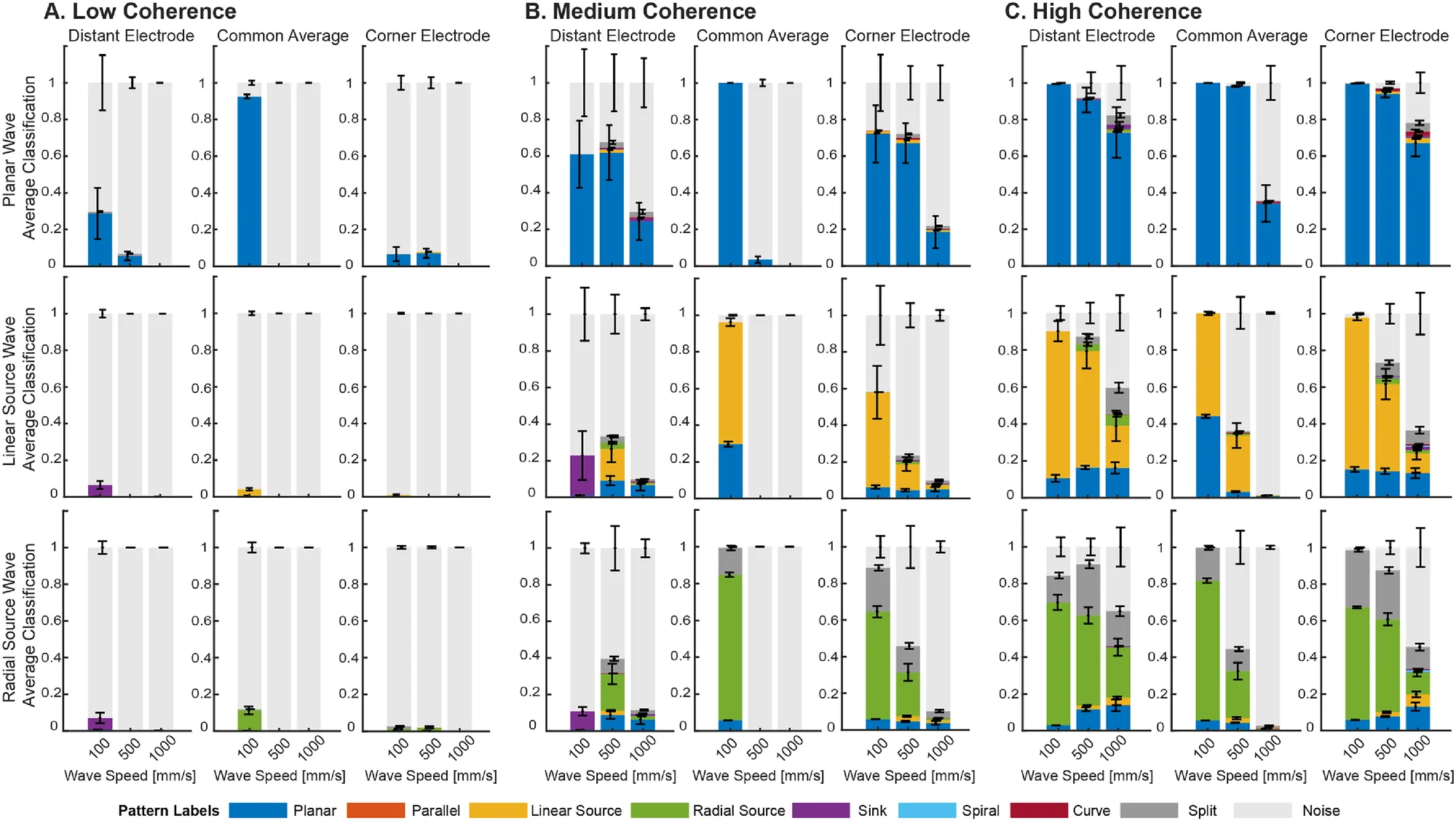
Using a corner electrode reference yields the most accurate wave pattern classification across conditions. Simulation results are shown for three coherence levels: (A) Level 3 (low), (B) Level 6 (medium), and (C) Level 8 (high). Simulated wave patterns were a planar wave (top row), linear source wave (middle row), and radial source wave (bottom row). For each condition, the classifications are shown for a distant electrode reference, common average reference, and corner electrode reference, averaged over 30 simulations of iEEG. Stacked bar plots indicate the mean classification percentages for each wave pattern, and error bars show the variance for each wave pattern across all simulations. For example, in the middle row of subfigure C, the prominent yellow bars indicate that a high percentage of subgrids were correctly classified as linear sources, but the smaller blue bars indicate that a percentage of subgrids were misclassified as planar waves. Perfect classification would be represented by a bar with a height of one, with blue bars for planar waves (top row), yellow bars for a linear source (middle row), and green bars for a radial source (bottom row). Abbreviation: iEEG = intracranial EEG.

When the iEEG coherence was low, the percentage of accurately classified propagation patterns was low for all referencing schemes (figure 4(A)). Only 100 mm s^−1^ planar waves could be reliably classified under these noisy conditions, when using the common average reference. Notably, at this low wave speed, common average referencing outperformed the other methods across most propagation patterns. However, at higher speeds (⩾500 mm s^−1^), the percentage of accurately classified patterns decreased to zero for all three patterns we tested. The performance of common average referencing at moderate-to-high wave speeds was similarly weak for medium coherence levels (figure 4(B)), and it resulted in lower rates of accurate detections in high coherence levels compared to the distant and corner references (figure 4(C)).

The distant electrode and corner electrode referencing schemes performed similarly overall, but each exhibited distinct strengths and weaknesses. At low coherence levels (figure 4(A)), patterns were predominantly classified as noise across both referencing schemes. However, low speed planar wave activity was more accurately classified when using a distant electrode reference. On the other hand, within the same low coherence setting, using a distant electrode reference led to the misclassification of source activity as a sink. At medium coherence levels (figure 4(B)), the accuracy of wave pattern classification improved for both single and corner electrode referencing schemes across all wave speeds. Notably, at 100 mm s^−1^, source activity was again misclassified when using the distant electrode reference, whereas the corner electrode reference showed the most consistent performance across all wave speeds. As coherence reached high levels (figure 4(C)), classification accuracy improved for the distant electrode reference, reaching levels comparable to the corner electrode reference.

Results for additional wave patterns are included as supplementary material (supplementary figure 3). The trend for sink patterns was similar to the source pattern results for the distant and corner electrodes. The corner electrode, again, showed consistency across wave speeds whereas use of a distant electrode reference led to misclassification of a sink as source activity for waves speeds of 100 mm s^−1^ in low and medium coherence scenarios. The spiral pattern was the most difficult to discern, with the most reliable estimates found in low wave speed conditions (100 mm s^−1^) across all referencing schemes (supplementary figure 3).

**Figure 5.**
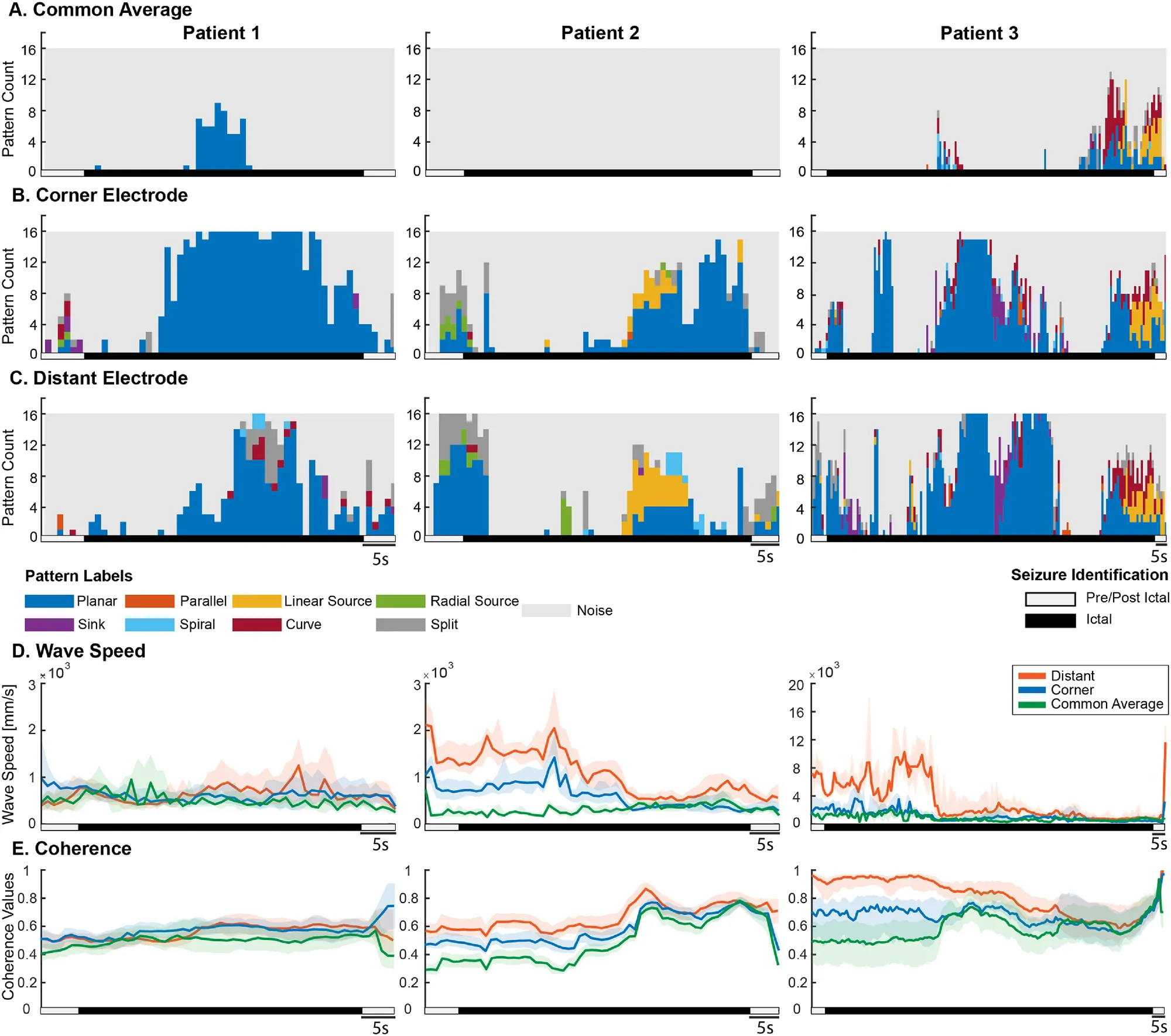
Effects of referencing schemes on wave pattern classification using human seizure data. Results are shown for one representative seizure from each of three different patients, with each column showing results from one patient. Each ictal iEEG recording was clipped to include 10 s before onset and 10 s after termination, where the horizontal black bar indicates the ictal period. Wave direction estimates were computed using three referencing schemes: (A) common average, (B) corner electrode, and (C) distant electrode reference. Stacked bar plots depict the distribution of classified wave patterns across all 16 subgrids in the 8 × 8 HD grid for all referencing schemes as a function of time, with each color representing a different wave pattern. The calculation was done on 10 s windows, shifted in time by 1 s. (D) Median wave speeds across all electrodes, with line color indicating the referencing scheme. Shaded regions represent the IQR. (E) Median coherence values across all electrodes, with line color indicating the referencing scheme. Shaded regions represent the IQR. Across referencing schemes, trends over time were similar; however, the distant electrode reference tended to have the highest coherence and wave speed values, and the common average reference tended to have the lowest values. Abbreviations: iEEG = intracranial EEG, IQR = interquartile range, HD = high-density.

The results when analyzing human iEEG were consistent with the simulation results (figure 5). Across all seizures, we found that the corner and distant electrode references performed similarly (figures 5(B and C)), with the corner electrode providing more sensitive pattern identification at lower coherence levels (figure 5(E)). The common average reference results lacked sensitivity, as patterns went undetected for large portions of the ictal periods (figure 5(A)). Overall, more patterns were detected when wave speeds were low (<500 mm s^−1^) and coherence levels were high (>0.5), which was more likely to occur as seizure termination neared (figures 5(D and E)).

Note that coherence and wave speed varied depending on the selected referencing scheme. Across referencing methods, the coherence followed similar trends over time, but it tended to be the highest for the distant electrode reference and lowest for the common average reference (figure 5(E)). The same was seen for wave speed, with the distant electrode yielding the highest values, followed by the corner and then common average reference (figure 5(D)).

One consideration when selecting a distant electrode as a reference is whether the electrode exhibits any epileptiform activity during the ictal period. For all three patients, we recalculated the wave pattern classification results using an alternative distant electrode reference that exhibited epileptiform discharges (supplementary figure 4). This choice of reference led to severely inflated coherence and different estimated wave patterns than the ones observed using quiet distant and corner references. For example, curve patterns were more frequent in Patient 1, split wave patterns were more frequent in Patient 2, planar wave patterns increased at termination for Patient 2, and no sink patterns were detected in the middle of Patient 3’s seizure. This emphasizes the importance of carefully selecting both the referencing method and the specific channel used for referencing.

### 3.3. Higher sampling rates yield higher pattern classification performance

**Figure 6.**
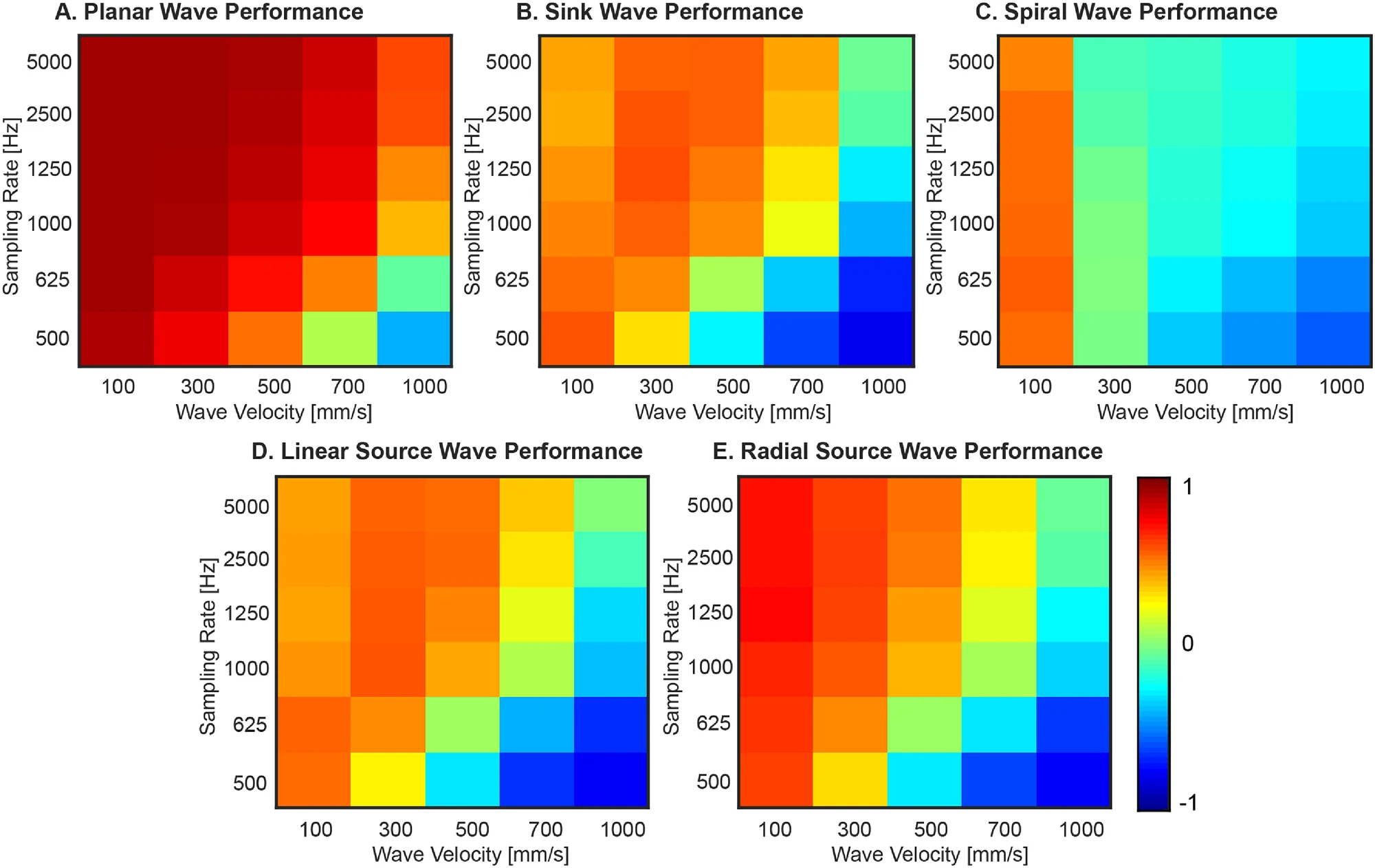
Effects of sampling rate and wave speed on wave direction estimation. The average classification performance values are shown as a function of sampling rate and wave velocities for (A) planar, (B) sink, (C) spiral, (D) linear source, and (E) a radial source. Averages were calculated across 30 iterations of each simulation, and heatmaps of the IQR values for each wave pattern are shown in supplementary figure 5. High wave speeds require high sampling rates for accurate classification, whereas wave speeds < 300 mm s^−1^ can be accurately classified at low sampling rates. Abbreviation: IQR = interquartile range.

We examined the relationship between sampling rate and wave pattern classification performance across different wave speeds using a high iEEG coherence that reflects the signal characteristics typically observed in most ictal events (figure 6, supplementary figure 5). Our results show that classification performance is dependent on wave speed and sampling rate, as higher wave speeds required higher sampling rates for accurate classification. At low wave speeds (⩽300 mm s^−1^), most wave patterns could be accurately classified for a sampling rate as low as 500 Hz (figures 6(A), (B), (D) and (E)). To accurately classify patterns at higher wave speeds, sampling rates ⩾1000 Hz were required. Planar waves had the highest pattern classification performance for the majority of wave speed and sampling rate combinations (figure 6(A)). Spiral waves were again the most difficult to measure, with accurate classification only occurring at the lowest wave speed (figure 6(C)).

It is important to note that classification performance across sampling rates is dependent on the overall coherence level. This was confirmed from various replicated experiments using medium iEEG coherence, in which we found that across all wave speeds, higher sampling rates were required to maintain a high classification performance (supplementary figure 6). Moreover, we found differences between downsampling the iEEG signals (keeping every *n*th sample of data) and using the resample method in MATLAB, which implements anti-aliasing methods to preserve low frequency activity [3]. When using the resample function, we found that all patterns could be equally accurately resolved at all sampling rates (supplementary figure 7). Instead, wave speed was the dominant factor in the ability to reliably estimate wave direction. This suggests that iEEG data could be recorded at a high sampling rate then subsequently resampled at a lower rate to reduce computation time without compromising the ability to estimate wave directions.

**Figure 7.**
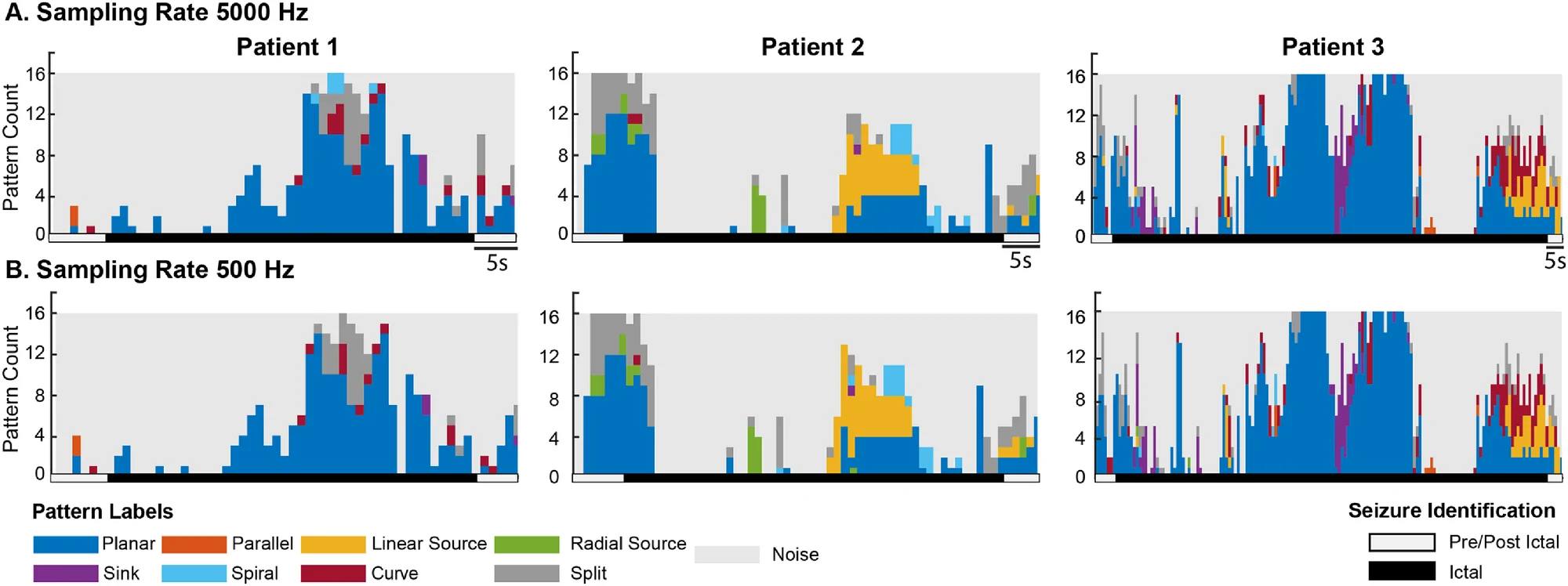
Effect of sampling rate on wave pattern classification in human ictal iEEG. We tested our ability to resolve wave directions at (A) 5000 Hz sampling rate and (B) after down sampling to 500 Hz, with data referenced to a quiet distant electrode. Each column represents results from one human subject. Stacked bar plots depict the distribution of classified wave patterns across all 16 subgrids in the 8 × 8 HD grid as a function of time, with each color representing a different wave pattern. The calculation was done on 10 s windows, shifted in time by 1 s. The original iEEG and downsampled iEEG produced very similar wave pattern classification results. Abbreviation: iEEG = intracranial EEG.

We then tested the impact of sampling rate on our ability to detect wave patterns in human iEEG (figure 7). We first measured wave patterns during the ictal period at the original sampling rate of 5000 Hz (figure 7(A)). The iEEG data were then downsampled to 500 Hz (figure 7(B)), after which we recalculated wave directions and reclassified the resulting patterns. We found that we were able to accurately estimate wave directions at 500 Hz, with only small variations in the resulting patterns compared to the 5000 Hz sampling rate, likely due to the high iEEG coherence during the ictal period (figure 5(E)). Results at 1000 Hz sampling rate were comparable to that of the 5000 Hz signals.

### 3.4. Increased electrode grid spacing compromises wave direction estimation accuracy

To assess the impact of electrode grid spacing on our ability to classify wave patterns, we tested progressively downsampled grids (3 mm, 6 mm, and 9 mm spacing) while keeping the wave velocity (500 mm s^−1^), and coherence level (8) constant. The 3 mm spacing matches the HD grid used for our iEEG recordings, and the 9 mm spacing approximates standard clinical subdural grid spacing. Here we use MSE to quantify the results instead of classification performance because each grid spacing has a different number of subgrids.

**Figure 8.**
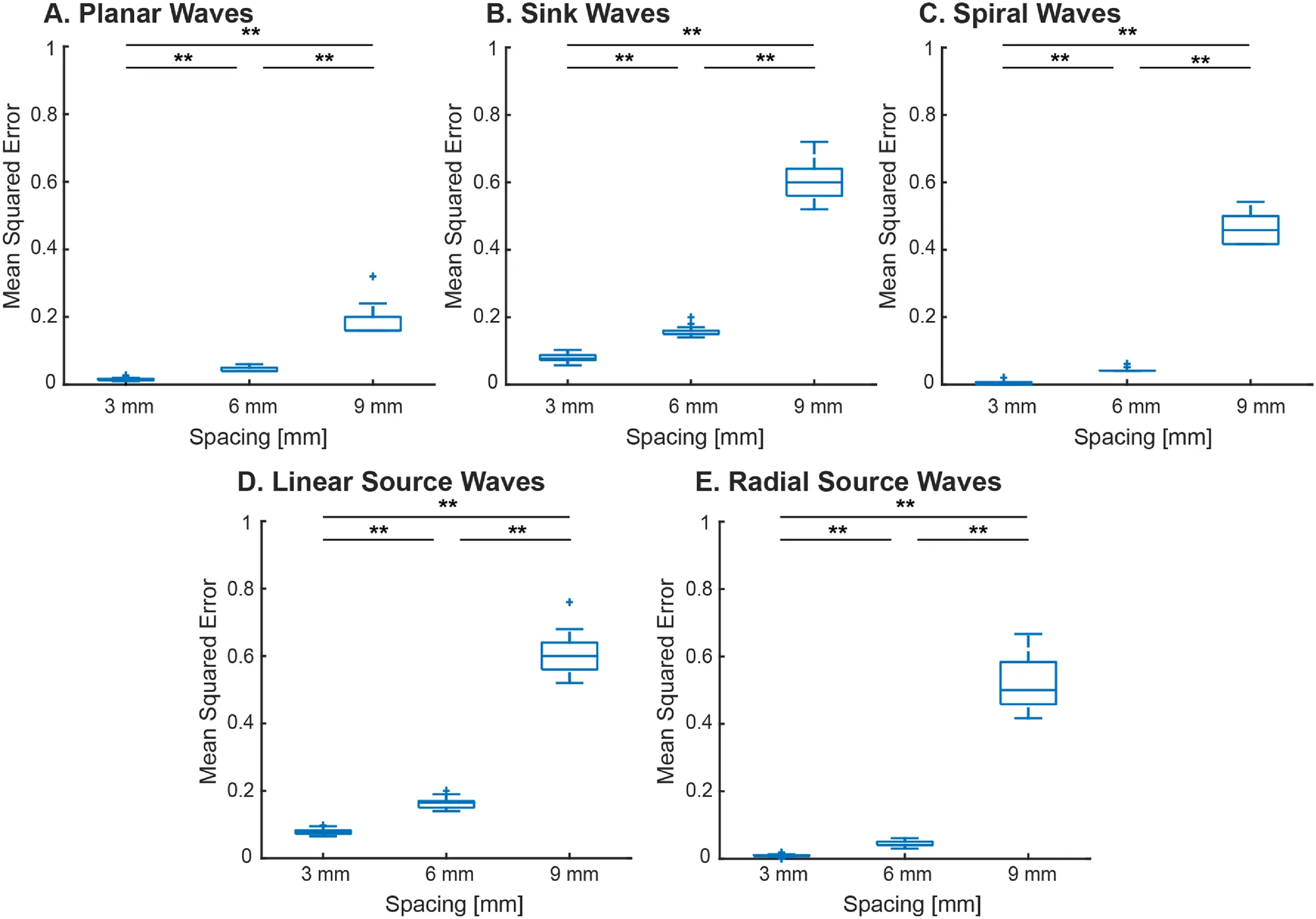
As the distance between electrodes increases, the error for estimating wave directions also increases. MSE for each simulated wave pattern using interelectrode distances of 3 mm, 6 mm, and 9 mm are shown. MSE was computed between the input wave pattern and the resulting measured wave directions for all three distances. Results are shown for (A) planar waves, (B) sink waves, (C) spiral waves, (D) linear source waves, and (E) radial source waves. Boxplots show the distribution of MSE values across 30 iterations of each simulation. Intracranial EEG data were simulated using coherence level 8 and wave velocity of 500 mm s^−1^, and significance was determined using the Wilcoxon signed rank test, where ** indicates *p* < 0.01. For all wave patterns, MSE for interelectrode distances of 3 mm and 6 mm was fairly low, but it increased significantly for electrodes spaced 9 mm apart. Abbreviation: MSE = mean-squared error.

As the grid spacing increased, the ability to resolve wave direction decreased for all wave patterns (*p* < 0.01), driving MSE values closer to 1 (figure 8). For the 9 mm spacing, the lowest MSE occurred for planar waves. We extended this analysis by testing all three referencing schemes, and a similar trend was observed across all patterns (supplementary figure 8). To further confirm our results, we analyzed iEEG seizure data from the implanted 8 × 8 macroelectrode grids for all 3 patients (electrode spacing = 10 mm). In contrast to the patterns detected using 3 mm spacing (figure 5), the estimated wave directions at 10 mm spacing resulted in patterns classified only as noise/unknown.

## 4. Discussion

In this study, we examined both inherent properties of human iEEG data and methodological factors that impact the measurement of ictal traveling waves. We developed a controlled simulation environment to systematically manipulate key variables including iEEG coherence, wave pattern types, referencing schemes, sampling rates, and grid spacing. This approach was motivated by the conflicting theories regarding ictal wave propagation; we hypothesized that variation in analytical techniques may have contributed to the differing conclusions in prior studies. Overall, our results demonstrate that careful assessment of iEEG characteristics and an informed selection of analysis methods are critical for optimizing pattern classification performance.

There are currently significant challenges in understanding seizure wave propagation [1–5, 24]. Traveling waves of electrical activity propagate quickly across the cortex during seizures, and the source of these waves is thought to be associated with the SOZ. However, there are competing theories about the nature of the source(s) [3]. One theory involves a slow propagating ictal wavefront, characterized as a long burst of high gamma power (80–150 Hz) that spreads across the cortex at approximately 1 mm s^−1^ [4–7]. This ictal wavefront has only been observed using microelectrode arrays and has been theorized to emit waves traveling at faster speeds (10–100 cm s^−1^) spreading longer distances across the cortex [4].

These fast traveling waves have been further investigated at both the micro and macro spatial scales which led to the development of a second source theory, which is that these waves are emitted by a static or moving radial source [1, 2, 25]. Recent studies attempting to rectify these disparate results suggest the involvement of a combination of these sources, each dominating seizure activity at different times [3]. Critically, variation in methodology across these studies (different electrode size and spacing, methods for time delay estimation, and sampling rates), may contribute to the lack of a unified theory.

We derived specific guidelines for robust wave direction estimation and pattern classification based on the results of our analysis (table 2). For high wave speeds (>500 mm s^−1^), higher levels of coherence are necessary for accurate wave pattern classification; if iEEG signals and their corresponding wave pattern do not meet these criteria, they can be excluded from further analysis or labeled with a lower level of confidence. In general, applying a common average reference resulted in a majority of subgrids and time points being classified as noise/unknown. Instead, we recommend referencing to an electrode at the corner of the subdural grid, as it enables reliable detection even in noisier conditions. A distant, single electrode reference can also be used, as it performs similarly to a corner reference in high coherence situations. However, its wave pattern classification performance is lower at medium levels of coherence, misclassification of source and sink waves is possible, and the results will vary depending on whether or not the reference channel exhibits epileptiform activity during the seizure. Regarding interelectrode spacing, our analysis suggests that distances of 6 mm or less enable accurate wave pattern classification, while spacing of 9 mm should be used with caution, especially for complex patterns beyond planar waves. Lastly, sampling rate had minimal impact for high coherence data; however, a sampling rate of at least 1000 Hz is recommended for robust and reliable analysis.

**Table 2.** A summary of the recommended methods and parameters for estimating wave direction, based on our results.

Factor	Recommendation
EEG coherence levels for low wave speeds ${ }( \unicode{x2A7D}\!500{\text{ mm}}\,{\mathrm{/}}\,{\mathrm{s}}$)	${\mathrm{Coherence}} \unicode{x2A7E}\!0.35$
EEG coherence levels for high wave speeds ($ &gt; 500{\text{ mm}}/{\mathrm{s}}$)	${\mathrm{Coherence}} \unicode{x2A7E}\!0.5$
Referencing scheme	Corner electrode
Sampling rate (assuming high coherence)	$ \unicode{x2A7E}\!1000{\text{ Hz}}$
Time delay estimation algorithm	Group delay
Grid size/spacing	At least $5\, \times \,5{\text{ with}} \unicode{x2A7D}\!6{\text{ mm}}$ spacing

Our results highlight the significant influence of referencing schemes on wave direction estimation. Prior research has shown that using a common single-electrode reference can artificially elevate coherence and induce phase synchronization [16], potentially distorting delay-based measures [15]. While common average referencing has been proposed to mitigate this by removing global signals from a distant reference, it may also eliminate meaningful seizure-related activity due to the high synchrony of ictal events [17, 18]. Our simulation results showed that wave speeds above 100 mm s^−1^ failed to be detected using a common average reference. On the contrary, the simulated distant and corner electrode references had comparable results, yielding higher pattern classification accuracies for wave speeds greater than 100 mm s^−1^.

When investigating these referencing schemes in human iEEG, we found results consistent with our simulations. For example, when implementing a common average reference, we found a reduction in coherence levels; thus, most patterns were classified as noise for most or all of the seizure across all patients. Although the quiet, distant reference increased overall coherence, its results were largely comparable to those of the corner reference, which maintained more moderate coherence levels. Additionally, we further evaluated the effects of referencing to a distant electrode that exhibited epileptiform activity (usually recruited later in the seizure), characterized by higher amplitude activity than the quiet reference. This approach led to severely elevated coherence values and the emergence of propagation patterns that were not present when using either the quiet distant or corner [19]. This finding is especially informative, as it is often challenging to identify a truly ‘quiet’ reference electrode. Our results therefore suggest that a corner electrode reference may serve as a reliable choice for wave direction analysis.

We found that accurate detection of fast propagating waves requires higher sampling rates. In prior studies utilizing microelectrode recordings, sampling rates were as high as 30 kHz and downsampled to a sampling rate as low as 500 Hz [1, 3, 4]. Our results demonstrate that accurate detection of fast propagating waves (>500 mm s^−1^) is dependent on the overall sampling rate. We show that when data is highly coherent and wave speeds are low, we are able to reproduce results with a sampling rate of 500 Hz that are consistent with those derived from our original 5000 Hz signal. However, higher sampling rates are needed for accurate detection of faster waves. Past studies implemented the resample method in MATLAB [3] as a means of downsampling the signal while preserving the low frequency components, which motivated us to test this method in our simulations. We found that applying the resampling method negated the impact of sampling rate, with accurate detection depending solely on wave speed. This can be useful for reducing computational time and for centers that have limited resources for data storage.

Electrode size and spacing directly impact our ability to reliably track wave propagation. Previous studies explored traveling waves at both micro (80 *µ*m diameter, 400 *µ*m spacing) and macro (2.4 mm diameter, 10 mm spacing) spatial scales [1–3]. While macroscale recordings offer broader cortical coverage, our results suggest they lack the spatial resolution necessary to reliably track wave direction. Through simulations of various electrode spacings, we found that grid densities with inter-electrode distances of ⩽6 mm were effective for capturing wave directions. However, this capability significantly deteriorated at 9 mm spacing, a trend further validated by our analysis of iEEG data from standard subdural grids in humans (10 mm spacing), which yielded no detectable wave patterns. While the results for interelectrode spacing will be a function of wave speed (in theory, small grid spacing with low wave speed should be equivalent to bigger grid spacing with higher wave speed), our simulations were based on typical ictal wave speeds reported in prior studies (100–1000 mm s^−1^) [1, 2, 4]. Overall, the HD subdural grid used in this study presents a promising compromise, offering expanded cortical coverage while preserving high spatial and temporal resolution; moreover, previous studies suggest that HD arrays can improve SOZ localization [26, 27]. However, even with this improved resolution, micro-scale dynamics such as the ictal wavefront could not be resolved, highlighting a continued need for finer-scale approaches to fully characterize seizure-related propagation phenomena.

There are several limitations to our study. When classifying our wave patterns, we developed a library of prominent wave patterns seen in the data, and this manual method may introduce some error, or several pattern variations may not have been accounted for. In cases where the linear sink and source models performed slightly worse at 100 mm s^−1^ than at 300 mm s^−1^ (figures 3(B, D) and figures 6(B, D), visual inspection revealed that wave directions near the center, where the nonlinearity occurs, could be recovered for the 100 mm s^−1^ wave speed. At higher wave speeds, no direction was estimated in this region, producing a slightly lower reported error due to the higher number of missing wave directions. While this issue was only seen in the linear source and sink patterns, future work should implement a more automated classification process that does not require a library of predeveloped patterns, to reduce sensitivity to noisy results. Moreover, when we measured the ability to accurately classify various wave patterns under different referencing schemes, we found that each method can lead to misclassifications at various wave speeds and coherence levels. When analyzing human iEEG data it can become difficult to discern what is a true pattern vs. potential misclassification under a particular referencing scheme. While we demonstrated the successful application of our method to human iEEG from three subjects, more rigorous methods for detecting and excluding spurious wave directions are needed, and the relationship between the ictal wave patterns and the SOZ should be investigated in a larger cohort of patients.

Furthermore, while our algorithm performs well within the desired range of wave speeds, accurate tracking at higher speeds depends on high signal coherence. Our tests used coherence levels typically observed during seizures, yet some seizures exhibit exceptionally high coherence, with an average >0.9. In these cases, the algorithm still identified wave patterns, though the effects of such high coherence and wave speed combinations remain unexplored. Finally, our study focused on investigating the strengths and weaknesses of the group delay algorithm as it has been used in multiple studies for wave direction analysis [1, 3], and is the most robust against noise. However, more work needs to be done to investigate the performance of other algorithms such as the max descent algorithm [3, 4] or a variation of the phase multilateration algorithm outlined in [2, 25].

## 5. Conclusion

This study identified the key factors influencing reliable estimation of ictal traveling waves in iEEG. Our results highlighted the critical roles of signal coherence and wave speed in shaping propagation estimates, and we demonstrated how these factors should inform methodological decisions. We provided practical guidelines for wave direction analysis, including recommendations for referencing scheme selection, electrode spacing, and sampling rate. These findings offer a foundation for more standardized and reproducible wave propagation studies, contributing to the broader goal of developing a unified theory of the sources underlying traveling waves in the brain. Ultimately, this has the potential to aid clinicians in localizing seizure generating tissue in patients with drug-resistant epilepsy, leading to better long-term outcomes in this population.

## Data Availability

The data that support the findings of this study are available upon reasonable request from the authors. The data being used here are a subset of a larger dataset being used for another, related paper. The full dataset will be published with that paper. Supplementary Material available at: https://doi.org/10.1088/1741-2552/ae9bed/data1.
